# Rad50 mediates DNA demethylation to establish pluripotent reprogramming

**DOI:** 10.1038/s12276-020-0467-0

**Published:** 2020-07-14

**Authors:** Hanseul Park, Byounggook Cho, Jongpil Kim

**Affiliations:** 1 Laboratory of Stem Cells & Gene Editing, Department of Biomedical Engineering, Dongguk University, Seoul, 100-715 Republic of Korea; 2 Department of Chemistry, Dongguk University, Seoul, 04620 Republic of Korea

**Keywords:** Reprogramming, Induced pluripotent stem cells

## Abstract

DNA demethylation is characterized by the loss of methyl groups from 5-methylcytosine, and this activity is involved in various biological processes in mammalian cell development and differentiation. In particular, dynamic DNA demethylation in the process of somatic cell reprogramming is required for successful iPSC generation. In the present study, we reported the role of Rad50 in the DNA demethylation process during somatic cell reprogramming. We found that Rad50 was highly expressed in pluripotent stem cells and that Rad50 regulated global DNA demethylation levels. Importantly, the overexpression of Rad50 resulted in the enhanced efficiency of iPSC generation via increased DNA demethylation, whereas Rad50 knockdown led to DNA hypermethylation, which suppressed somatic cell reprogramming into iPSCs. Moreover, we found that Rad50 associated with Tet1 to facilitate the DNA demethylation process in pluripotent reprogramming. Therefore, our findings highlight the novel role of Rad50 in the DNA demethylation process during somatic cell reprogramming.

## Regenerative medicine: Protein boosts stem cell generation process

Heightened expression of a DNA repair protein improves efficiency when generating induced pluripotent stem cells (iPSCs) for use in regenerative medicine. DNA demethylation – the removal of methyl groups from one of the DNA bases, cytosine – is required for effective reprogramming of cells other than sperm and egg cells to create iPSCs. Ineffective demethylation has been a key challenge for scientists to overcome in generating iPSCs efficiently. Now, Jongpil Kim and co-workers at Dongguk University in Seoul, South Korea, have demonstrated that the DNA repair protein Rad50 plays a regulatory role in DNA demethylation during cell reprogramming. Rad50 interacts with a key enzyme involved in demethylation, boosting the efficiency of the process. The team found that overexpressing Rad50 increased DNA demethylation during reprogramming, enhancing the efficiency of iPSC generation. Blocking Rad50 had the opposite effect.

## Introduction

DNA methylation is a major gene regulatory mechanism that is essential for mammalian development^1,2^. In mammals, 5-methylcytosine (5mC) is a major epigenetic modification that is generated by the addition of a methyl group to cytosine nucleotides in DNA by DNA methyltransferase (DNMT) family members (DNMT1, DNMT2, DNMT3A, DNMT3B, and DNMT3L)^3,4^. DNA methylation gradually increases during development, and there are different methylation profiles for each cell type; however, DNA methylation is stable once cells are fully differentiated^5^. For example, over 98% of DNA methylation occurs in the CpG island of somatic cells. However, there is no DNA methylation in embryonic stem cells (ESCs)^4^.

In contrast, DNA demethylation is the process of removing methyl groups from the cytosines of DNA^6^. Demethylation of cytosines in DNA can be achieved through either passive or active mechanisms^6,7^. The passive demethylation of DNA is known to be mediated by the loss of methyl groups in cytosine over several rounds of replication, whereas active demethylation is mainly mediated by 10–11 translocation (TET) enzymes that oxidize 5mC to produce 5-hydroxymethylcytosine (5hmC)^8^. DNA demethylation also plays an essential role in early mammalian development. For example, a previous study showed that preimplantation embryos experience a loss of methylation in the early stage of development^8^. In addition, abnormal DNA demethylation has been closely associated with genomic imprinting-related diseases, cancer, and mental disorders^9^.

Recent studies of somatic cell reprogramming revealed that extensive demethylation of DNA in somatic cells is essential for epigenetic reprogramming^10,11^. Several lines of evidence suggest that DNA demethylation may play a critical role in reactivating pluripotency genes^12^. Thus, increased DNA demethylation can lead to efficient reprogramming. For example, a DNA methyltransferase inhibitor was reported to improve the reprogramming progress^13^. In addition, vitamin C was found to enhance reprogramming efficiency by demethylation of 5mC^14^. Consistent with these findings, tet methylcytosine dioxygenase 1 (Tet1) can replace Oct4 during the reprogramming process because it promotes demethylation through 5hmC conversion in the methylated region of Oct4^15^. However, despite the importance of DNA demethylation in the process of somatic cell reprogramming, the mechanisms that control DNA demethylation in this process remain largely unknown.

In this study, we demonstrate that Rad50 can play an important role in active DNA demethylation during somatic cell reprogramming. Previously, Rad50 was reported to be a key player in DNA double-strand break repair (DSBR) in MRN complexes^16^. Rad50 is highly conserved and belongs to the ABC transporter family of ATPases. The ATPase activity of Rad50 is known to be essential for MRN function by inducing conformational changes in the MRN complex^17,18^. However, previous studies have shown that the DNA repair mechanism is closely implicated in active DNA demethylation^7,19–23^. For example, DNA repair factors such as growth arrest and DNA damage-inducible protein 45 alpha (Gadd45a) are involved in the DNA demethylation process, which erases methylated markers to facilitate DNA repair^24^. Therefore, these results proposed a possible mechanism mediated by Rad50, which could link both the DNA demethylation process and the DNA repair process.

In this study, we identified the unexpected role of Rad50 in DNA demethylation in the cell reprogramming process. We found that Rad50 was exclusively expressed in pluripotent ES and iPS cells. Importantly, Rad50 overexpression resulted in the demethylation of DNA in differentiated fibroblasts, which ultimately enhanced reprogramming efficiency. Moreover, Rad50 was required for the DNA demethylation process in somatic cell reprogramming. We further demonstrated the role of Rad50 as a Tet1-binding protein to promote active DNA demethylation, which could induce 5hmC. Our study provides new insights into the control of the DNA demethylation process in establishing pluripotency during reprogramming.

## Materials and methods

### Cell culture

For mouse embryonic fibroblasts (MEFs), the cells were maintained in DMEM supplemented with 10% FBS and 1% Pen/Strep (100 U/mL penicillin and 100 μg/mL streptomycin) at 37 °C and 5% CO_2_ in a humidified incubator. The cells were passaged every 3–5 days with trypsin (Sigma). Mouse iPSCs (miPSCs) and mouse ESCs (mESCs) were maintained on 0.2% gelatin-coated plates in culture medium consisting of DMEM/F12 supplemented with 15% FBS (Invitrogen), 1 mM l-glutamine (Invitrogen), 1% nonessential amino acids (Invitrogen), 1% penicillin-streptomycin, 0.1 mM β-mercaptoethanol (Sigma), LIF (Millipore), and with or without doxycycline at 37 °C and 5% CO_2_ in a humidified incubator. Cells were passaged every 3–5 days. For OSKM-mediated cellular reprogramming, MEFs were infected with TetO-FUW-OSKM and FUW-M2rtTA viruses. Subsequently, the viruses were removed, and the day on which doxycycline was added to the ESC medium was defined as day 0 postinfection. The cells tested negative for mycoplasma with a MycoSensor PCR Assay Kit (Agilent) every 3 months by STR analysis serviced by Kogene Biotech. The experimenter was not blinded to the treatment. None of the experiments were excluded from our analyses.

### Immunocytochemistry

Immunohistochemical analysis was performed on mouse cells. The cells were washed with 1× PBS followed by 4% paraformaldehyde. Postfixed cells were washed three times and then were blocked for 30 min. Then, the cells were incubated with primary antibodies overnight at 4 °C. We used the following primary antibodies: rabbit anti-Nanog (1:250, ab80892; Abcam) and mouse anti-Oct4–3/4 (c-10) (1:250, sc-5279; Abcam). After incubation, the cells were washed three times for 5 min each with PBS. Then, samples were incubated with the appropriate secondary antibody at room temperature for 1 h. The cells were washed three times for 5 min with PBS. Samples were counterstained with 4′,6-diamidino-2-phenylindole (DAPI, 1:1000; Invitrogen, Carlsbad, CA, USA) for 5 min at room temperature. Subsequently, they were imaged with a Zeiss LSM 800 confocal microscope (Carl Zeiss, Thornwood, NY, USA) and Eclipse Ti-U microscope (Nikon, Tokyo, Japan). The experimenter was not blinded to the treatment. None of the experiments were excluded from our analyses.

### Western blotting

For western blotting, cells were gently washed with 1× PBS. RIPA buffer (1% NP-40, 0.5% DOC, 0.1% SDS, and 150 mmol/L NaCl in 50 mmol/L Tris, pH 8.0; Sigma-Aldrich) and 1× proteinase inhibitor were added to the cells, which were then homogenized. Western blot loading buffer (5×) was added to the samples, and they were boiled at 100 °C for 10 min. The samples were separated by 12% sodium dodecyl sulfate-polyacrylamide gel electrophoresis (SDS-PAGE). Then, the separated samples were blotted onto a membrane. The membrane was incubated with primary antibodies overnight at 4 °C. We used the following primary antibodies: rabbit anti-Rad50 (1:1000, ab89; Abcam), rabbit anti-Oct4 (1:1000, ab18976; Abcam), rabbit anti-methylcytosine dioxygenase (TET1) (1:1000, 09–872; Millipore), and rabbit anti-β-actin (1:1000, LF-PA0207; Abfrontier). The bands were visualized with an ECL kit (DG-WF200; Dogen). The experimenter was not blinded to the treatment. None of the experiments were excluded from our analyses.

### Real-time PCR analysis

Total RNA was isolated using an eCube Tissue RNA Mini Kit (Philekorea) according to the manufacturer’s instructions. The isolated RNA (1 µg) was reverse-transcribed using AccuPower® CycleScript RT PreMix (Bioneer) for cDNA synthesis. Quantitative PCR analysis was performed using AccuPower® PCR PreMix (Bioneer) with primers. The reaction was carried out using a Rotor-Gene Q real-time PCR machine (Qiagen).

### Dot blot assay

Genomic DNA was extracted following standard procedures. The sample DNA (2 µL) was transferred to a nitrocellulose membrane and blocked with 5% BSA in TBST. The membrane was incubated overnight at 4 °C with primary antibodies, mouse anti-5-methylcytosine (5mC) (1:1000, ab73938; Abcam) and rat anti-5-hydroxymethylcytosine (5hmC) (1:1000, ab106918; Abcam), to detect 5hmC and 5mC following the manufacturer’s protocol.

### Bisulfite sequencing

For bisulfite sequencing analysis, total genomic DNA was isolated using an eCube Tissue DNA Mini Kit (Philekorea) according to the manufacturer’s instructions. The genomic DNA (2 µg) was modified via sodium bisulfite conversion of unmethylated cytosines using an EpiTect Bisulfite Kit (Qiagen) following the manufacturer’s recommended protocol. Bisulfite-modified genomic DNA was amplified using PCR primers designed by the PrimerSuite website (www.primer-suite.com). The PCR-amplified products were then purified with a NucleoSpin® Gel and PCR Clean-up kit (Macherey-Nagel) and were cloned using the TA Cloning™ Kit (Thermo Scientific) for bisulfite sequencing.

### Alkaline phosphatase staining

Alkaline phosphatase (AP) staining was performed using an Alkaline Phosphatase Detection Kit (Millipore) according to the manufacturer’s instructions. In brief, the cells were washed three times with 1× PBS followed by 4% paraformaldehyde for 5 min. Postfixed cells were washed three times with 1× PBS. Then, the cells were incubated with fast red stain and naphthol AS-MX at room temperature for 10 min.

### Coimmunoprecipitation

For coimmunoprecipitation, cells were first gently washed with cold 1× PBS to remove the media. Harvested cells were lysed using RIPA buffer containing a proteinase inhibitor cocktail. Following sonication, the samples were incubated with G protein-coupled magnetic beads (Thermo) for 1 h to remove nonspecific binding material. Then, the samples were incubated with anti-FLAG (Sigma), anti-Tet1 (09–872; Millipore), or anti-Rad50 (ab89, Abcam) antibodies overnight at 4 °C. The beads were washed with RIPA buffer and eluted for western blotting.

### Bioinformatics analysis

Methyl-CpG-binding domain sequencing (MBD-seq) was performed as described^25^. In brief, DNA was fragmented by binding to the methyl-CpG-binding domain. DNA fragments were sequenced using a HiSeq instrument (Illumina). RNA sequencing was performed following a reported protocol^26^. Briefly, total RNA was extracted with an eCube Tissue RNA Mini Kit (PhileKorea) and sequenced by an Illumina instrument. ChIP sequencing (ChIP-seq) was performed following a reported protocol^27^. A ChIP-grade rabbit anti-Rad50 antibody (1:1000, ab89; Abcam) was used for sequencing. MBD-seq, RNA-seq, and ChIP-seq were performed by Genomictree, Inc. (Daejeon, Korea).

## Results

### Rad50 binding to methylated Oct4 promoter

To identify novel proteins that might be involved in demethylating DNA in cells that are in a pluripotent state, we initially attempted to detect proteins that specifically bind to the methylated Oct4 promoter in mouse ESCs (mESCs). In brief, methylated DNA sequences derived from the Oct4 promoter region were conjugated to biotin beads, and they were incubated with nuclear protein extracts derived from mESCs. Then, proteins bound to the methylated DNA sequences were identified by mass spectrometry (Supplementary Fig. 1a, b). We identified several protein candidates that were bound to the methylated DNA sequences, and Rad50 was one of the major identified binding proteins (Supplementary Table 1).

**Fig. 1:**
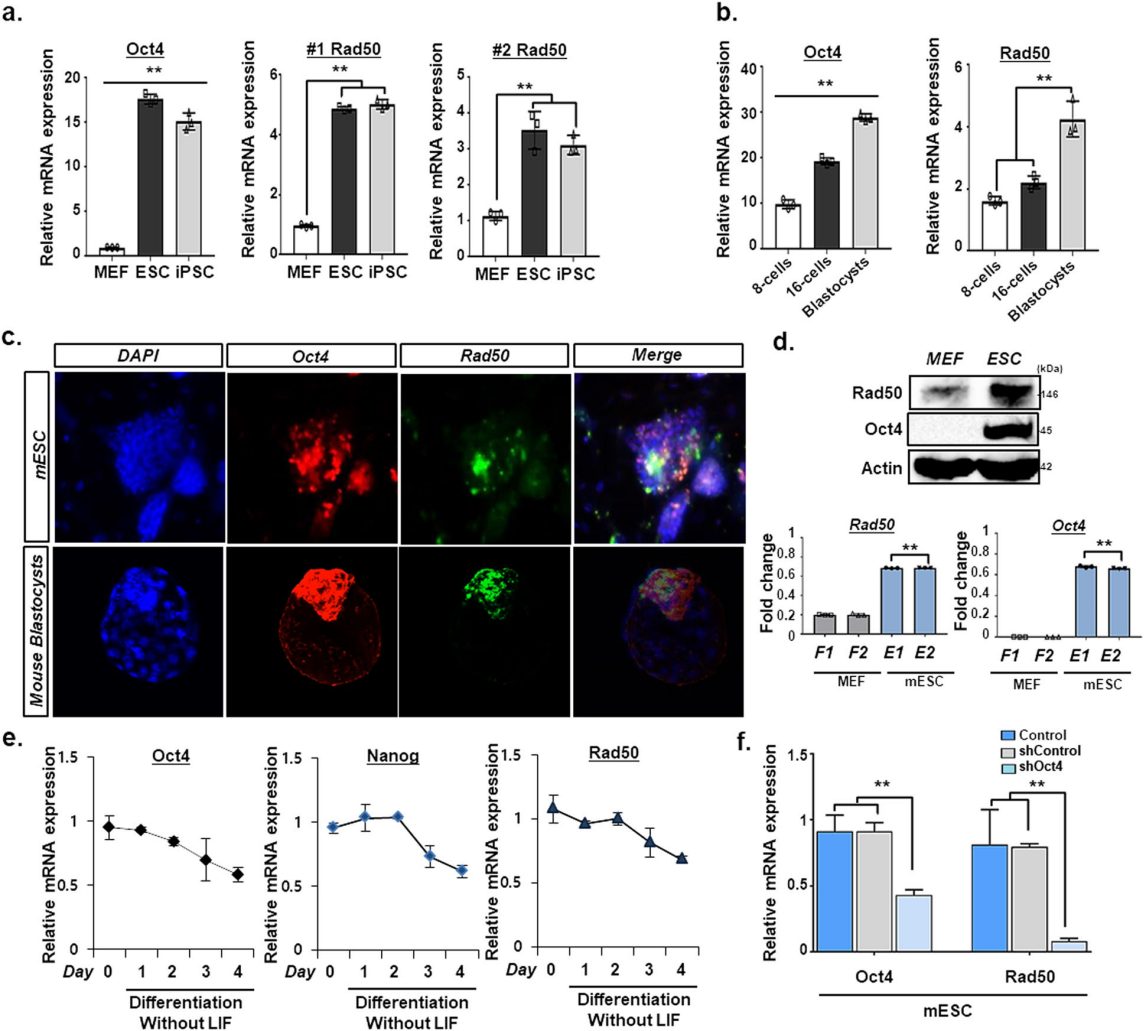
Expression of Rad50 in pluripotent cells. **a** Quantitative real-time PCR analysis of Oct4 and Rad50 in MEFs, mESCs, and miPSCs. Data are expressed as the mean ± SEM. **p < 0.01, ANOVA with Tukey’s post hoc test. **b** Quantitative real-time PCR analysis of Oct4 and Rad50 at the 8-cell stage, 16-cell stage, and E3.5 blastocyst-stage mouse embryos. Data are expressed as the mean ± SEM. **p < 0.01, ANOVA with Tukey’s post hoc test. **c** Immunohistochemistry for Oct4 (Red), Rad50 (green), and DAPI (blue) in mESCs and blastocyst-stage embryos. **d** Western blot analysis for Rad50 and Oct4 in MEFs and mESCs. **e** Quantitative real-time PCR analysis of Oct4, Nanog, and Rad50 expression in mESCs following LIF withdrawal. **f** Quantitative real-time PCR analysis of Oct4 and Rad50 in mESCs following the addition of siOct4. Data are expressed as the mean ± SEM. **p < 0.01, ANOVA with Tukey’s post hoc test. The images (**c**, **d**) are representative of ≥3 similar experiments.

To determine the role of Rad50 in DNA demethylation of cells in the pluripotent state, we first examined the expression of Rad50 in ESCs, iPSCs, and mouse embryonic fibroblasts (MEFs). Notably, we found that Rad50 was exclusively expressed in ESCs and iPSCs that maintain low DNA methylation levels (Fig. 1a and Supplementary Fig. 2a). Additionally, we observed an increase in the expression of other DNA repair genes, Mre11, Nbs1, Brca1, and Rad51, in mESCs and iPSCs (Supplementary Fig. 2b). However, other DNA binding proteins were not specific to the pluripotent state (data not shown). Importantly, Rad50 expression was significantly increased in the development of 8-cell stage mouse embryos to the blastocyst stage, where DNA methylation levels are known to be the lowest in mammalian development (Fig. 1b–d). Consistent with this result, Rad50 was significantly downregulated during differentiation induced by LIF withdrawal, which was closely correlated with the pluripotent markers Oct4 and Nanog (Fig. 1e). Moreover, we found that Rad50 was significantly downregulated following the knockdown of Oct4 in ESCs (Fig. 1f), suggesting that Rad50 might play a role in the regulation of pluripotency via DNA demethylation.

**Fig. 2:**
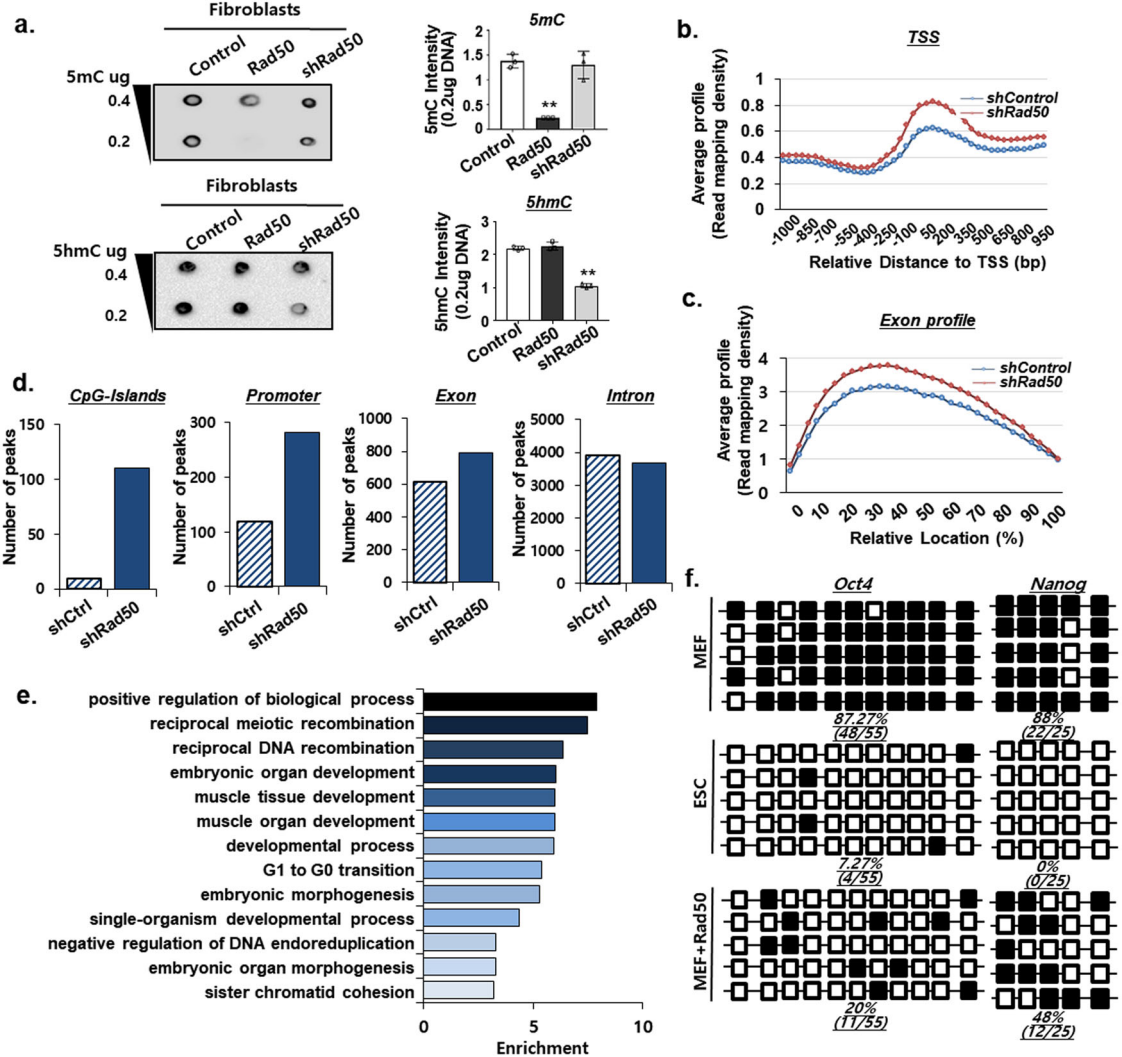
Rad50 induction of global DNA demethylation. **a** Dot blot analysis of 5mC and 5hmC in diluted genomic DNA from MEFs with Rad50 overexpression or knockdown. The image is representative of ≥3 similar experiments. **b** Average profile read mapping densities at the TSS determined by MBD-seq. **c** Average profile read mapping densities at the exon determined by MBD-seq. **d** Annotation of the differentially hypermethylated CpG islands, promoter, exon, and intron region following Rad50 overexpression or knockdown. **e** Gene ontology (GO) enrichment analysis of the hypermethylated region following knockdown of Rad50. **f** Bisulfite sequencing of the Oct4 and Nanog promoter regions in MEFs, mESCs, and Rad50-overexpressing MEFs.

Next, to determine the role of Rad50 in DNA demethylation, we monitored the levels of 5mC and 5hmC in fibroblasts that overexpress or have depleted levels of Rad50. Importantly, we observed that the overexpression of Rad50-induced global DNA demethylation, whereas the inhibition of Rad50 reduced DNA demethylation, decreasing 5hmC levels (Fig. 2a). Moreover, methyl-CpG-binding domain sequencing (MBD-seq) confirmed the hypermethylation of genomic DNA in the CpG island, promoter, TSS, and exon regions following Rad50 inhibition (Fig. 2b–d). Gene ontology (GO) enrichment analysis of MBD-seq data identified biological processes related to DNA recombination or developmental process, indicating that Rad50 was involved in biological processes related to DNA repair or cell fate conversion (Fig. 2e). Moreover, to confirm demethylation, we assessed the effects of DNA methylation on the pluripotency genes Oct4, Nanog, and Tbx15 by bisulfite sequencing analysis. The methylation pattern of the CpGs of Oct4 and Nanog promoters showed that the CpGs were predominantly methylated in fibroblasts, and the CpGs in ESCs showed complete methylation, which was consistent with what was observed in previous studies. However, Rad50 overexpression in fibroblasts induced DNA demethylation and significantly reduced methylation levels (Fig. 2f). Taken together, our results indicated that Rad50 was highly expressed in pluripotent cells and led to DNA demethylation in the promoters of pluripotency genes.

### Rad50 enhancement of epigenetic reprogramming efficiency of mouse somatic cells

**Fig. 3:**
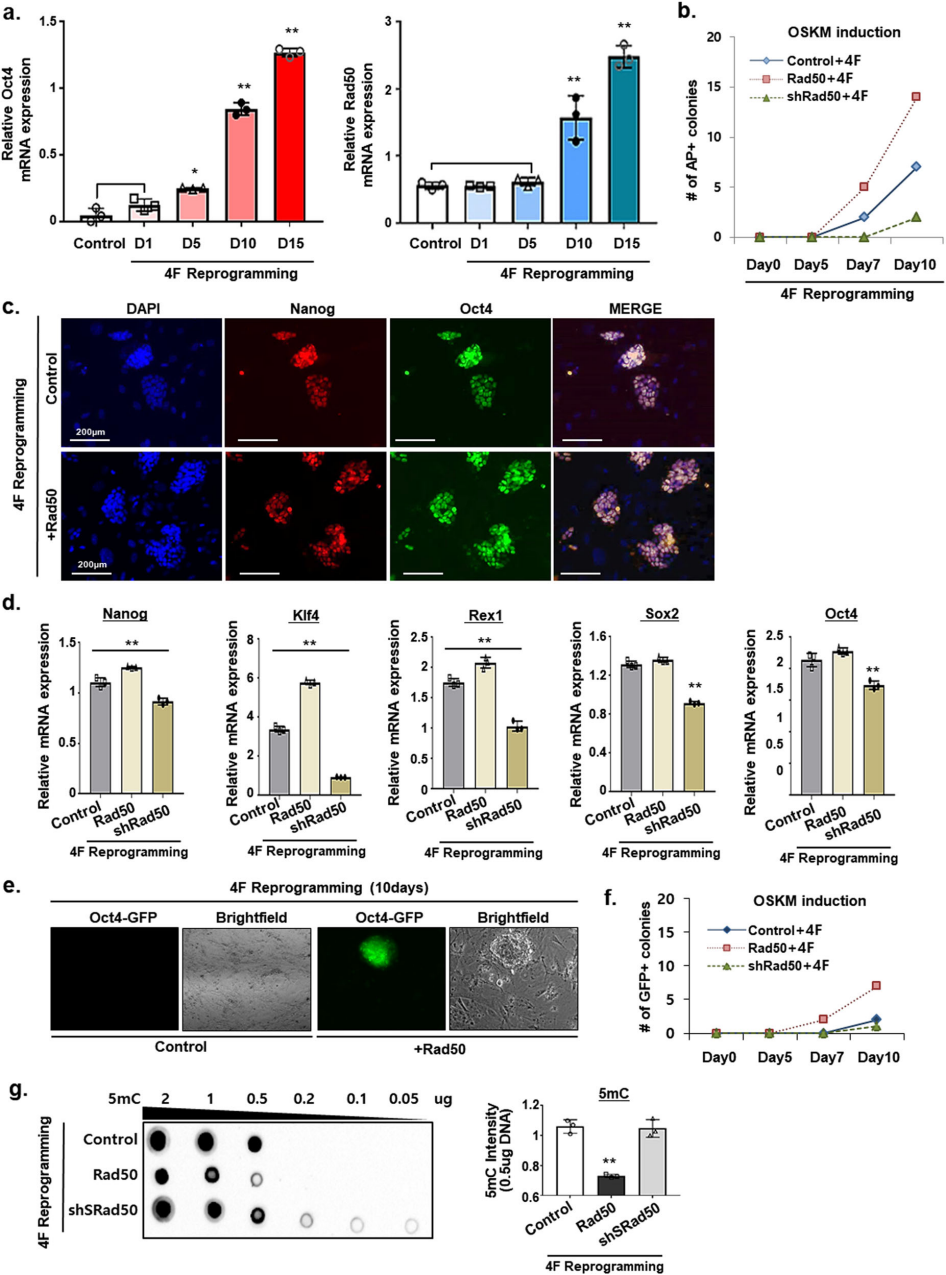
Rad50 enhancement of OSKM-induced reprogramming efficiency. **a** Quantitative real-time PCR analysis of Oct4 and Rad50 expression during OSKM-mediated somatic cell reprogramming. Data are expressed as the mean ± SEM. *p < 0.05 and **p < 0.01, ANOVA with Tukey’s post hoc test. **b** Number of AP + colonies after OSKM-mediated somatic cell reprogramming. **c** Immunohistochemistry for Nanog (red), Oct4 (green), and DAPI (blue) following Rad50 overexpression or knockdown during OSKM reprogramming. **d** Quantitative real-time PCR analysis of pluripotent markers. Data are expressed as the mean ± SEM. **p < 0.01, ANOVA with Tukey’s post hoc test. **e** Morphology and fluorescence profiles of Oct4-GFP fibroblasts overexpressing Rad50. **f** The number of Oct4-GFP-positive colonies was counted during OSKM reprogramming. **g** Dot blot analysis of 5mC in diluted genomic DNA from cells exhibiting Rad50 overexpression or knockdown at day 15 during OSKM reprogramming. Data are expressed as the mean ± SEM. **p < 0.01, ANOVA with Tukey’s post hoc test. The images (**c**, **e**, and **g**) are representative of ≥3 similar experiments.

Previously, it has been reported that DNA methylation is a major epigenetic barrier in cell reprogramming^28^, and the inhibition of DNA methylation is known to increase OSKM-mediated reprogramming efficiency^15^. Therefore, to determine the role of Rad50 in cell reprogramming, we first assessed the expression level of Rad50 during cell reprogramming. Endogenous Oct4 and Rad50 levels gradually increased during reprogramming and reached a maximum at 2 weeks after 4-factor induction (Fig. 3a). Moreover, of conditions tested, Rad50 overexpression resulted in the most efficient iPSC formation, whereas Rad50 knockdown inhibited iPSC generation, as confirmed by AP+, Oct4, and Nanog staining (Fig. 3b, c). Consistent with these results, the highest induction of Nanog, Oct4, Rex1, Sox2, and Klf4 was observed at day 15 after 4-factor induction (Fig. 3d). However, Rad50 inhibition led to a significant reduction in the expression of these genes (Fig. 3b–d). Moreover, efficient cell reprogramming was observed in Oct4-GFP knock-in (KI) fibroblasts following the overexpression of Rad50, where Oct4-GFP-positive colonies started to appear within 10 days (Fig. 3e, f). Additionally, we found that Rad50 combined with 4-factor expression significantly decreased 5mC levels, whereas increased 5mC levels were observed under 4-factor-induced conditions with Rad50 inhibition (Fig. 3g). These results demonstrated that efficient reprogramming mediated by Rad50 may be attributed to increased DNA demethylation. Finally, we examined whether DNA repair efficiency could be enhanced by 4-factor reprogramming. We found increased DNA repair efficiency in 4-factor reprogramming but did not observe enhanced DNA repair efficiency by Rad50 overexpression combined with 4-factor reprogramming (Supplementary Fig. 3a, b).

### Binding pattern similarity between Rad50 and Tet1

**Fig. 4:**
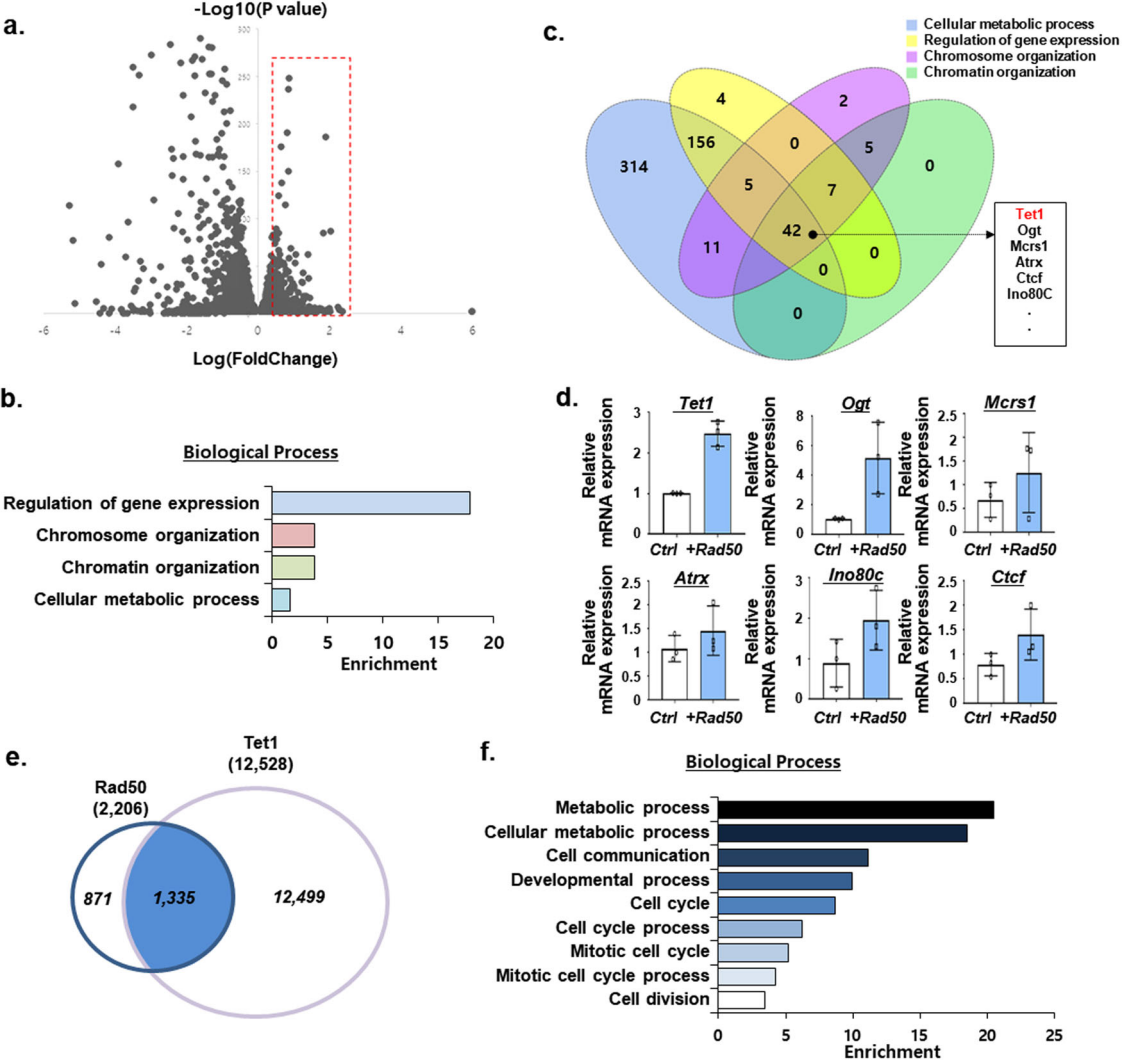
Rad50 interactions with Tet1. **a** Volcano plot reveals genes upregulated or downregulated by Rad50 overexpression, as determined by RNA-seq analysis. The threshold for determining differentially upregulated gene expression is indicated by a red box (Log(P value) > 2, Log(FoldChange) > 0.3). **b** GO enrichment analysis of significant upregulation as a result of Rad50 overexpression, as determined by RNA-seq analysis. **c** Venn diagram of the distribution of GO categories, cellular metabolic process, regulation of gene expression, chromosome organization, and chromatin organization, in Fig. 4b. **d** Quantitative real-time PCR validation of selected genes detected by RNA-seq analysis. Data are expressed as the mean ± SEM, *n* = 3. *p < 0.05 and **p < 0.01, two-sided Student’s *t* test. **e** Venn diagram showing overlapping binding regions shared between Rad50 and Tet1, as determined by Chip-seq analysis. **f** GO enrichment analysis of overlapping binding regions shared between Rad50 and Tet1.

To further investigate how Rad50 contributes to cell reprogramming, we compared the transcriptome of Rad50-overexpressing fibroblasts with that of control fibroblasts. Rad50 overexpression resulted in significant changes in global gene expression profiles. We identified 944 genes whose expression was significantly increased after Rad50 overexpression (Fig. 4a). GO enrichment analysis indicated that the upregulated transcripts were enriched for various biological processes, such as regulation of gene expression, chromosome organization, chromatin organization, and cellular metabolic process (Fig. 4b). To analyze the genes in each of the GO analyzed biological processes, a Venn diagram was utilized, and it identified the genes that were affected by Rad50 overexpression in the different biological processes. A total of 42 genes in Rad50 overexpression conditions were identified (Fig. 4c and Supplementary Table 2), and these genes were further validated by quantitative RT-PCR. Figure 4d shows the significant induction of Tet1, Ogt, Mcrs1, Atrx, Ino80c, and Ctcf in Rad50-overexpressing cultures (Fig. 4d). Interestingly, among the upregulated genes, Tet1 was one of the most significantly upregulated after Rad50 overexpression (Fig. 4a–c). Taken together, these results strongly suggest that Rad50-induced Tet1 expression and that Rad50 may be closely associated with Tet1 in the process of DNA demethylation. Next, we compared the genome-wide binding distribution of Rad50 and Tet1 by chromatin immunoprecipitation coupled with high-throughput sequencing (ChIP-seq). Figure 4e shows that most of the binding sites of Rad50 overlapped with those of Tet1. GO analysis of these binding sites identified the metabolic process, cellular metabolic process, cell communication, and developmental process (Fig. 4f). Moreover, ChIP-qPCR analysis showed the cooccupancy of Rad50 and Tet1 at the Oct4 and Nanog promoters, suggesting that the Rad50 and Tet1 interaction was the general mechanism of DNA demethylation in the process of somatic cell reprogramming (Supplementary Fig. 4).

Moreover, we examined whether Rad50 directly binds to Tet1. When endogenous Tet1 was pulled down, coimmunoprecipitation (Co-IP) with endogenous Rad50 was observed. In addition, endogenous Tet1 was coimmunoprecipitated when endogenous Rad50 was pulled down, suggesting that endogenous Rad50 could form a protein complex with Tet1 (Supplementary Fig. 5a, b). In addition, we examined whether the Rad50-Tet1 interaction was dependent on the Rad50-associated DNA repair system. The Tet1-Rad50 interaction was significantly decreased upon Mre11 and Nbs1 knockdown (Supplementary Fig. 6a, b), indicating that Rad50-Tet1 interaction was dependent on the expression of DNA repair genes.

### Role of Rad50 in Tet1-mediated DNA demethylation

**Fig. 5:**
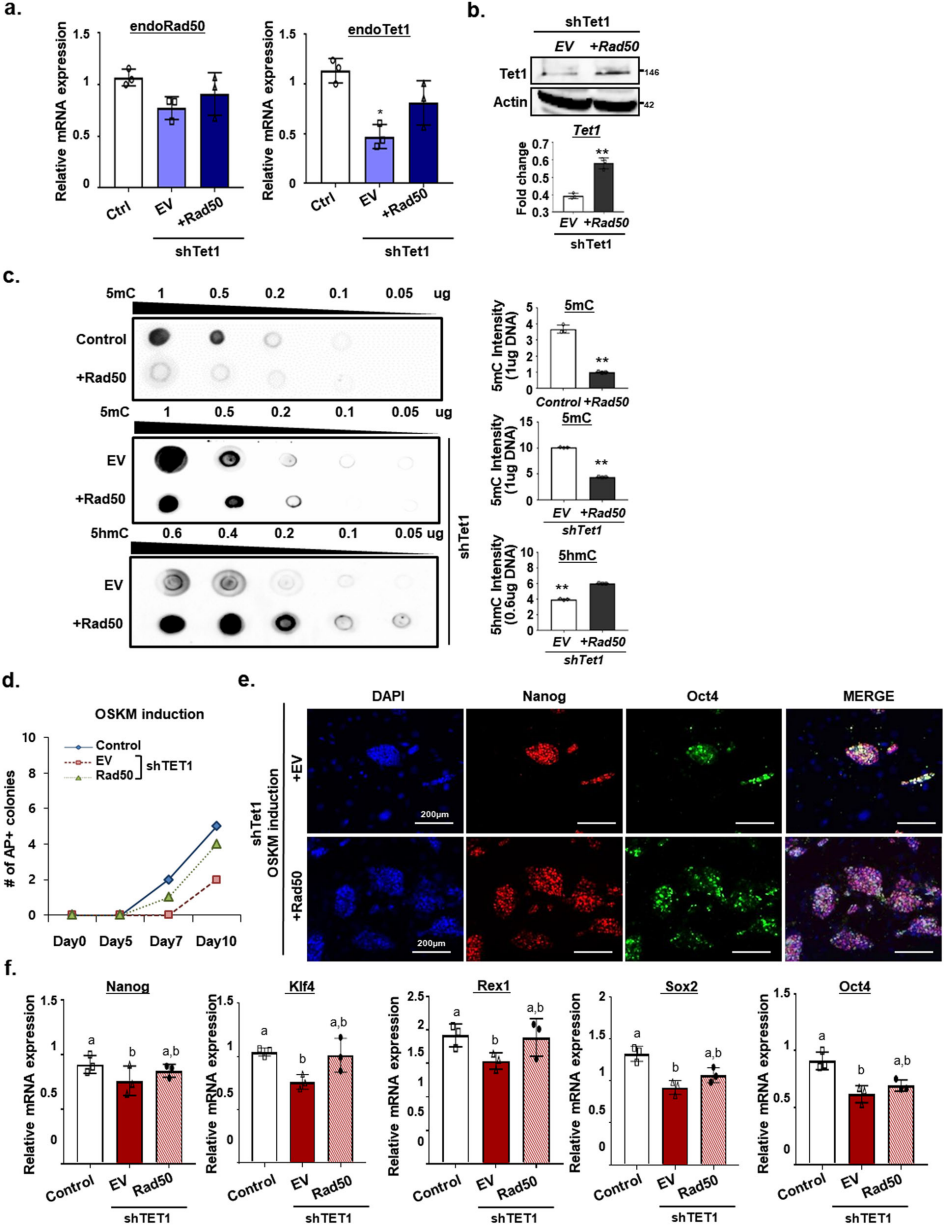
Tet1 regulation via Rad50. **a** Quantitative real-time PCR analysis of Tet1 and Rad50 after Tet1 knockdown in mESCs overexpressing Rad50 or treated with an EV (empty vector). Data are expressed as the mean ± SEM. *p < 0.05, ANOVA with Tukey’s post hoc test. **b** Western blot analysis of Tet1 and Oct4 after Tet1 knockdown in mESCs overexpressing Rad50. The bottom panel shows quantification of western blot results. Data are expressed as the mean ± SEM, *n* = 3. **p < 0.01, two-sided Student’s *t* test. **c** Dot blot analysis of 5mC and 5hmC in diluted genomic DNA from Tet1 knockdown mESCs that overexpress Rad50. The left panel shows the quantification of dot blot results. Data are expressed as the mean ± SEM, *n* = 3. **p < 0.01, two-sided Student’s *t* test. **d** The number of colonies was counted during OSKM reprogramming after Tet1 knockdown in fibroblasts with Rad50 overexpression or treated with an EV. **e** Immunohistochemistry for Nanog (Red), Oct4 (green), and DAPI (blue) after Tet1 knockdown in fibroblasts with Rad50 overexpression or treated with an under OSKM induction conditions. **f** Quantitative real-time PCR analysis of pluripotency-associated gene markers. Data are expressed as the mean ± SEM. **p < 0.01, ANOVA with Tukey’s post hoc test. The images (**b**, **c**, and **e**) are representative of ≥3 similar experiments.

To further determine whether Rad50 induces DNA demethylation with Tet1 activity, we assessed the effects of Rad50 expression after Tet1 silencing in mESCs. Quantitative RT-PCR analysis revealed that Rad50 overexpression in Tet1-silenced mESCs increased the expression of endogenous Rad50 and Tet1, indicating that Rad50 can induce Tet1 expression (Fig. 5a, b). Additionally, we confirmed the expression of DNA repair genes in Tet1 knockdown cells. Tet1 knockdown led to a decrease in the expression of DNA repair genes similar to what was observed in the Rad50 knockdown conditions (Supplementary Fig. 6c). Moreover, we evaluated the levels of 5hmC and 5mC in Rad50-overexpressing mESCs after silencing Tet1. Consistent with the previous results, dot blot analysis showed a decrease in 5mC following Rad50 overexpression (Fig. 5c, top panel). However, the increased 5mC levels in Tet1-depleted mESCs were sharply reduced by the overexpression of Rad50 (Fig. 5c, middle panel). Moreover, the decreased 5hmC levels in Tet1-depleted mESCs were also upregulated by the overexpression of Rad50 (Fig. 5c, lower panel), suggesting that Rad50 can rescue Tet1-mediated DNA demethylation.

To further confirm that Rad50 regulation of pluripotency is dependent on Tet1, we examined iPSC formation following the overexpression of Rad50 in 4-factor-transduced reprogrammed cultures with Tet1 knocked down. Consistent with previous results, Tet1 knockdown led to significant suppression of reprogramming efficiency^29,30^. However, the overexpression of Rad50 significantly rescued the phenotypes associated with Tet1 inhibition during reprogramming, generating iPSC colonies (Fig. 5d, e). Consistent with these results, we confirmed that Rad50 overexpression promotes the expression of pluripotent genes in reprogramming cultures with the inhibition of Tet1 (Fig. 5f). Taken together, these results suggest that Rad50-induced active DNA demethylation may rescue Tet1-mediated DNA methylation and that Rad50-mediated active DNA demethylation may play a critical role in the process of pluripotent reprogramming.

## Discussion

In mammalian development, epigenetic modifications play an important role in the cell fate decision process. Specifically, as a critical epigenetic modification in the genome, DNA methylation exhibit a dynamic pattern from early embryogenesis to various biological processes^1,7,9^. Moreover, recent studies have shown that DNA demethylation is dynamic during cell reprogramming and that incomplete DNA demethylation can be an obstacle to somatic cell reprogramming. Therefore, it is important to understand the DNA demethylation process to enable efficient cell reprogramming in regenerative medicine. Most recently, active DNA demethylation has been recognized as a specific epigenetic activity that opposes DNA methylation to regulate various biological processes. Active DNA demethylation involves multiple steps, and several candidate proteins are involved in the active DNA demethylation process. Tet1 has been proposed to be a key enzyme involved in the active demethylation of 5mC to produce 5hmC. Moreover, several active DNA demethylation mechanisms involving activation-induced cytidine deaminase (AID)/catalytic polypeptide-like (APOBEC) family members in which 5-methylcytosine deaminase results in thymine were previously reported^7,8,31^.

In this study, we identified an unexpected role of Rad50 protein as a key regulator of DNA demethylation in the process of somatic cell reprogramming. Importantly, Rad50 was required in DNA demethylation for efficient somatic cell reprogramming. Moreover, we observed phenotypes in which Rad50 overexpression increased the conversion of 5mC to 5hmC, promoting somatic cell reprogramming along with Tet1 activity. Moreover, we found that Rad50 interacted with a key enzyme of the DNA demethylation machinery (Tet1) and shared common targets across the genome that were bound by Tet1.

**Fig. 6:**
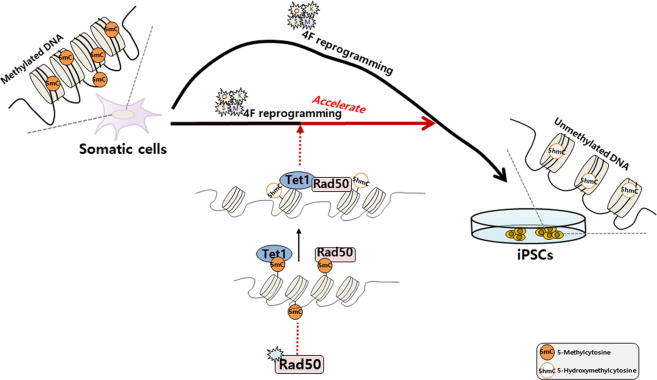
Schematic representation of Rad50 enhancement of somatic cell reprogramming. The interaction between Rad50 and Tet1 forms a positive feedback loop in the regulation of demethylation, which could enhance somatic cell reprogramming.

Previously, Rad50 proteins have been implicated in the biological process of DNA repair. Various studies have been conducted to understand the molecular mechanism underlying Rad50 activity in terms of DNA repair. For example, Rad50 forms a highly conserved complex with MRE11 and NBS1 at DNA double-strand breaks that plays a central role in genomic instability. Interestingly, knockout studies suggest that Rad50 is required for the survival of proliferative cells but is dispensable for the viability of postmitotic nondividing cells. Consistent with these results, we also found that Rad50 was exclusively expressed by pluripotent ESCs or iPSCs, indicating that Rad50 has an unknown role in undifferentiated stem cells that have high DNA demethylation levels. Moreover, in the present study, we found that Rad50 could preferentially bind to methylated DNA, and the genome-wide DNA methylation profile revealed Rad50-mediated DNA demethylation (Supplementary Fig. 1b), indicating that Rad50 acts to promote DNA methylation. Moreover, we showed that the unique binding sites of Rad50 are mostly coordinated with Tet1, which may be critical for inducing the conversion of 5hmC in the cell reprogramming process. Thus, in the present study, we propose that Rad50 with Tet1 could play an important role in epigenetic regulation for establishing pluripotent reprogramming (Fig. 6). Furthermore, we found that Rad50-mediated DNA demethylation can rescue Tet1-mediated demethylation activity. Previously, Tet1 has also been reported to play an important role in DNA repair and regulate DNA repair genes in mammalian cells^32^. Therefore, our study consistently demonstrated that the DNA repair mechanism involving Rad50 might be functionally linked to the DNA demethylation process. Consistent with these results, a previous study reported that active demethylation by Gadd45a occurs by DNA repair through the DNA repair endonuclease XPG^24,33^, suggesting that Gadd45a, a protein involved in DNA repair, may also play a key role in active DNA demethylation. Taken together, these results suggest a possible Rad50-mediated mechanism that links both DNA demethylation and DNA repair.

However, it is unclear whether Rad50 mediates the conversion of 5hmC in a locus-specific fashion during cell reprogramming. Further characterization of the binding sites of Rad50 with Tet1 in cell reprogramming will contribute to a better understanding of the mechanisms of DNA demethylation during cell reprogramming. In addition, DNA demethylation has been indicated to play an important role in reactivating pluripotency genes, suggesting that Rad50 may regulate the reactivation of pluripotency genes, which could be of interest in future investigations.

## Supplementary information

merged supplementary figures, and tables

## References

[1] Zeng, Y. & Chen, T. DNA methylation reprogramming during mammalian development. *Genes*10.3390/genes10040257 (2019). 10.3390/genes10040257 PMC6523607 30934924

[2] Li C (2018). DNA methylation reprogramming of functional elements during mammalian embryonic development. Cell Discov..

[3] Piccolo FM, Fisher AG (2014). Getting rid of DNA methylation. Trends Cell Biol..

[4] Jin B, Li Y, Robertson KD (2011). DNA methylation: superior or subordinate in the epigenetic hierarchy?. Genes Cancer.

[5] Meissner A (2008). Genome-scale DNA methylation maps of pluripotent and differentiated cells. Nature.

[6] Wu H, Zhang Y, Reversing DNA (2014). methylation: mechanisms, genomics, and biological functions. Cell.

[7] Bhutani N, Burns DM, Blau HM (2011). DNA demethylation dynamics. Cell.

[8] Kohli RM, Zhang Y (2013). TET enzymes, TDG and the dynamics of DNA demethylation. Nature.

[9] Wu SC, Zhang Y (2010). Active DNA demethylation: many roads lead to Rome. Nat. Rev. Mol. Cell Biol..

[10] Bhutani N (2010). Reprogramming towards pluripotency requires AID-dependent DNA demethylation. Nature.

[11] Takahashi K, Yamanaka S (2006). Induction of pluripotent stem cells from mouse embryonic and adult fibroblast cultures by defined factors. Cell.

[12] Hochedlinger K, Plath K (2009). Epigenetic reprogramming and induced pluripotency. Development.

[13] Mikkelsen TS (2008). Dissecting direct reprogramming through integrative genomic analysis. Nature.

[14] Chen J (2013). Vitamin C modulates TET1 function during somatic cell reprogramming. Nat. Genet..

[15] Gao Y (2013). Replacement of Oct4 by Tet1 during iPSC induction reveals an important role of DNA methylation and hydroxymethylation in reprogramming. Cell Stem Cell.

[16] Trujillo KM, Yuan SS, Lee EY, Sung P (1998). Nuclease activities in a complex of human recombination and DNA repair factors Rad50, Mre11, and p95. J. Biol. Chem..

[17] Lamarche BJ, Orazio NI, Weitzman MD (2010). The MRN complex in double-strand break repair and telomere maintenance. FEBS Lett..

[18] Uziel T (2003). Requirement of the MRN complex for ATM activation by DNA damage. EMBO J..

[19] Gehring M, Reik W, Henikoff S (2009). DNA demethylation by DNA repair. Trends Genet..

[20] Choi Y (2002). DEMETER, a DNA glycosylase domain protein, is required for endosperm gene imprinting and seed viability in arabidopsis. Cell.

[21] Jost JP (2001). 5-Methylcytosine DNA glycosylase participates in the genome-wide loss of DNA methylation occurring during mouse myoblast differentiation. Nucleic Acids Res..

[22] Gong Z (2002). ROS1, a repressor of transcriptional gene silencing in Arabidopsis, encodes a DNA glycosylase/lyase. Cell.

[23] Weiss A, Keshet I, Razin A, Cedar H (1996). DNA demethylation in vitro: involvement of RNA. Cell.

[24] Barreto G (2007). Gadd45a promotes epigenetic gene activation by repair-mediated DNA demethylation. Nature.

[25] Harris RA (2010). Comparison of sequencing-based methods to profile DNA methylation and identification of monoallelic epigenetic modifications. Nat. Biotechnol..

[26] Kunz M (2018). RNA-seq analysis identifies different transcriptomic types and developmental trajectories of primary melanomas. Oncogene.

[27] Wu S (2018). SWI/SNF catalytic subunits’ switch drives resistance to EZH2 inhibitors in ARID1A-mutated cells. Nat. Commun..

[28] De Carvalho DD, You JS, Jones PA (2010). DNA methylation and cellular reprogramming. Trends Cell Biol..

[29] Wei T (2015). An HDAC2-TET1 switch at distinct chromatin regions significantly promotes the maturation of pre-iPS to iPS cells. Nucleic Acids Res..

[30] Wang T (2013). Subtelomeric hotspots of aberrant 5-hydroxymethylcytosine-mediated epigenetic modifications during reprogramming to pluripotency. Nat. Cell Biol..

[31] Nabel CS (2012). AID/APOBEC deaminases disfavor modified cytosines implicated in DNA demethylation. Nat. Chem. Biol..

[32] Zhong J (2017). TET1 modulates H4K16 acetylation by controlling auto-acetylation of hMOF to affect gene regulation and DNA repair function. Nucleic Acids Res..

[33] Niehrs C, Schafer A (2012). Active DNA demethylation by Gadd45 and DNA repair. Trends Cell Biol..

