# Supplementary material for: Rad50 mediates DNA demethylation to establish pluripotent reprogramming
Source: Exp Mol Med. 2020 Jul 14;52(7):1116–27. doi: 10.1038/s12276-020-0467-0 (PMC8080709; doi:10.1038/s12276-020-0467-0)
Supplement: Supplementary file 1 — merged supplementary figures, and tables [file 12276_2020_467_MOESM1_ESM.docx]

**
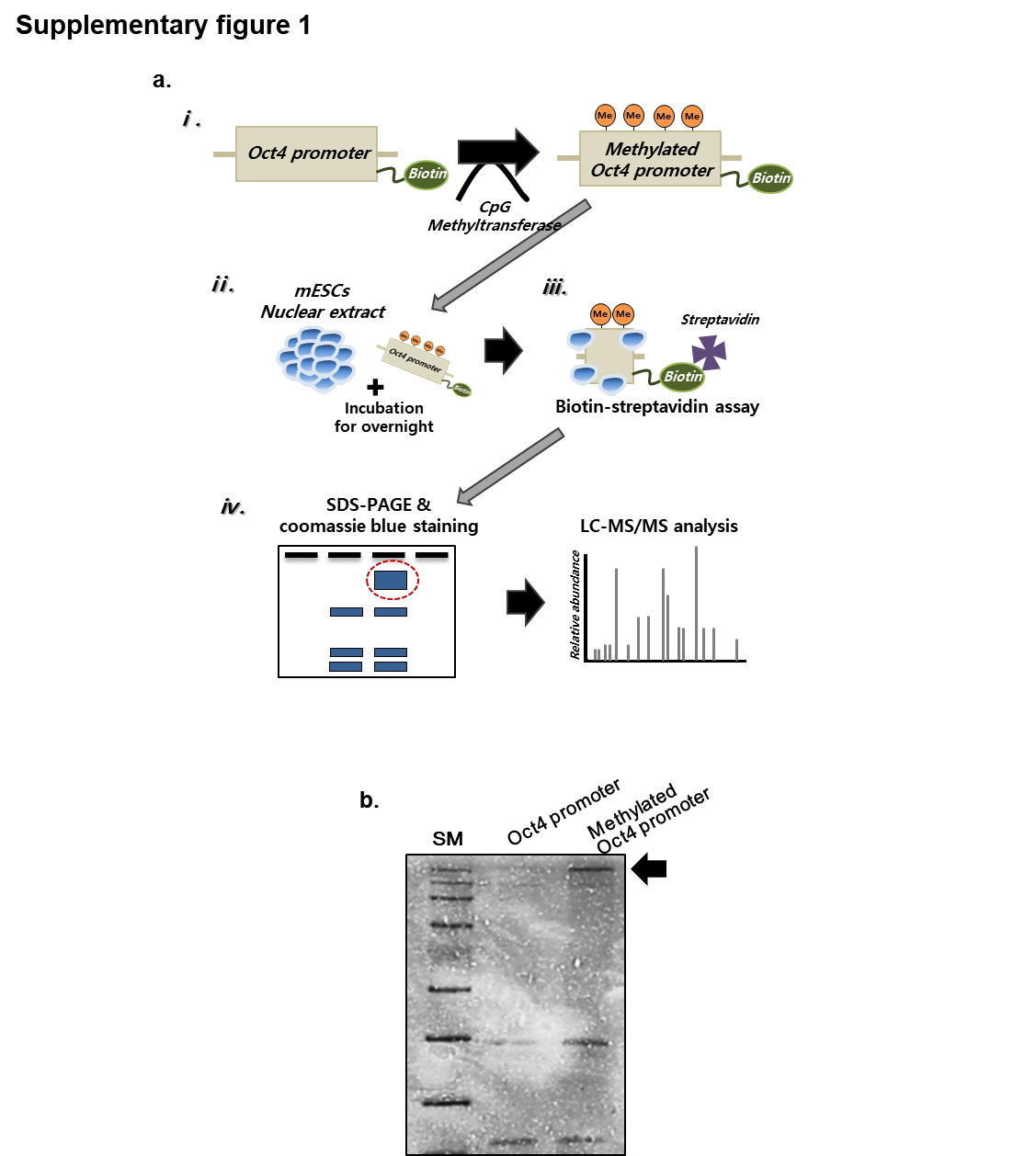
**

**Supplementary Figure 1 Identifying proteins that bind to methylated Oct4 promoter.** a. A flow chart of the screening process for identifying proteins that bind to the methylated DNA sequence. i) The Oct4 promoter fragment was conjugated with biotin beads. ii) After methylation or unmethylation of the Oct4 promoter fragment, biotin-labeled DNA-beads were incubated with nuclear extracts derived from mESCs. iii) Protein samples (methylated and unmethylated) were collected by streptavidin precipitation, separated, and further identified by mass spectrometry. b. Protein samples were separated on an SDS-PAGE gel. Lane of the sample, indicated by arrows, was cut and analyzed by LC-MS/MS. SM; size marker.

**
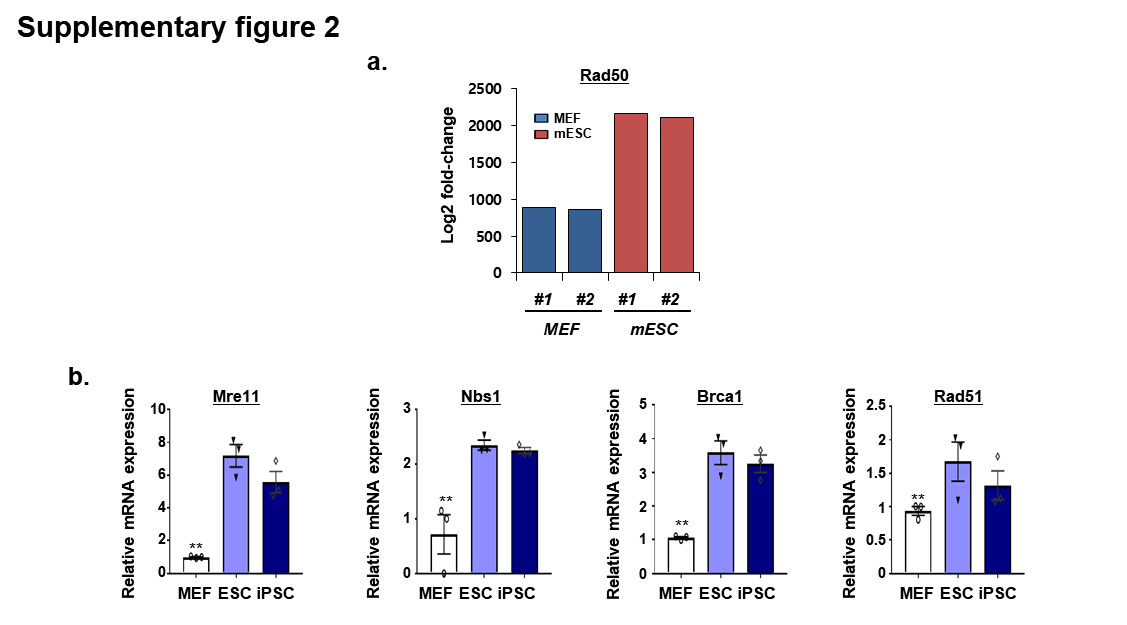
**

**Supplementary Figure 2 High Rad50 and DNA repair genes expression in pluripotent cells.** a. Rad50 fold-change values of RNA-seq data comparing MEFs and mESCs. RNA-seq datasets for MEFs and mESCs were obtained from a public database (GSE36290). b. Quantitative real-time PCR analysis of DNA repair genes (Mre11, Nbs1, Brca1, and Rad51) in MEFs, mESCs, and miPSCs. Data are expressed as the mean ± SEM. ***p* < 0.01, ANOVA with Tukey's post-hoc test.

**
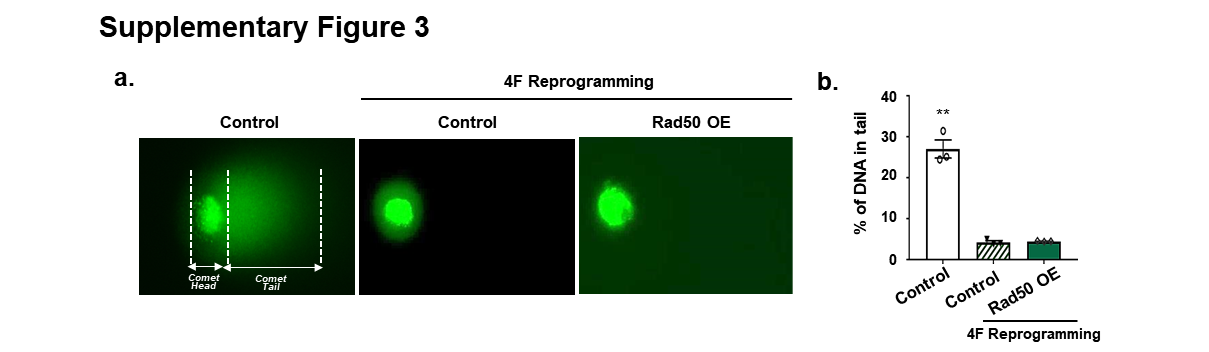
**

**Supplementary Figure 3 DNA repair efficiencies in 4 factors-induced pluripotent stem cells**. a. Comet assay represented as tail moment on MEF and 4 factors-induced reprogrammed of MEF. b. percentage of DNA in tail in Supplementary Fig.xa. Data are expressed as the mean ± SEM. ***p* < 0.01, ANOVA with Tukey's post-hoc test.

**
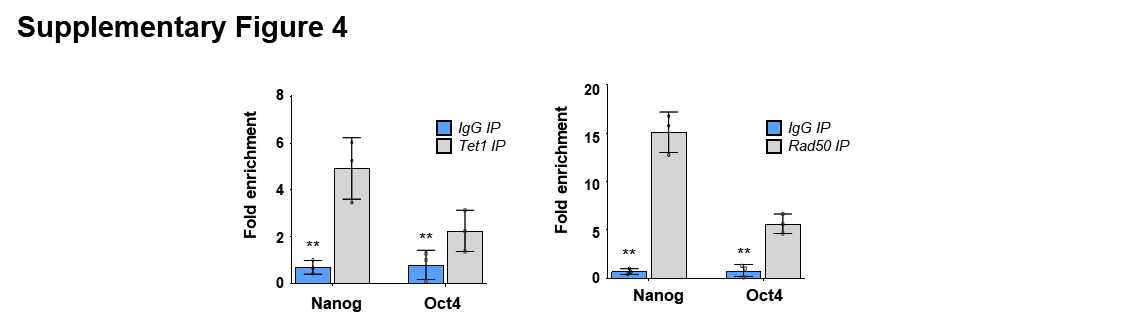
**

**Supplementary Figure 4 Tet1 and Rad50 interact with pluripotent genes.** ChIP-qPCR analysis for Tet1 and Rad50 enrichment at the promoter of representative pluripotent genes (Oct4 and Nanog) were performed.

**
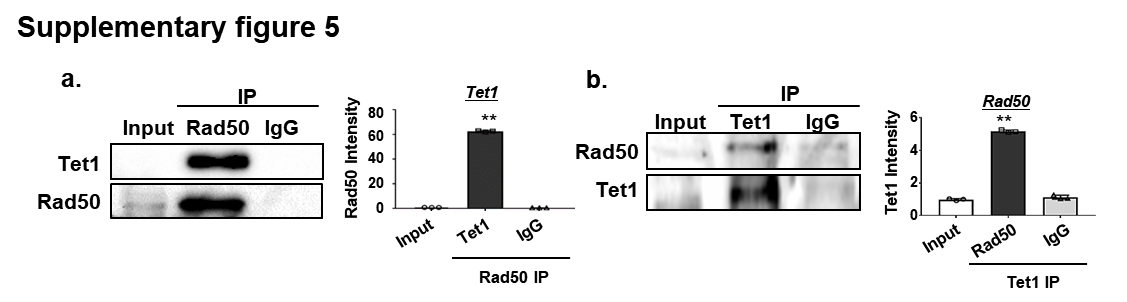
**

**Supplementary Figure 5 Epigenetic signatures of Rad50.** a. Co-immunoprecipitation (Co-IP) of Rad50 pulled down with Tet1. Cell lysates were processed for immunoprecipitation with anti-FLAG and anti-Rad50 and immunoblotted for Tet1. Data are expressed as the mean ± SEM. ***p* < 0.01, ANOVA with Tukey's post-hoc test. b. Co-IP of Tet1 pulled down with Rad50. Cell lysates were processed for immunoprecipitation with anti-FLAG and anti-Tet1 and immunoblotted for Rad50. Data are expressed as the mean ± SEM. ***p* < 0.01, ANOVA with Tukey's post-hoc test. The images (a, and b) are representative of ≥ 3 similar experiments.

**
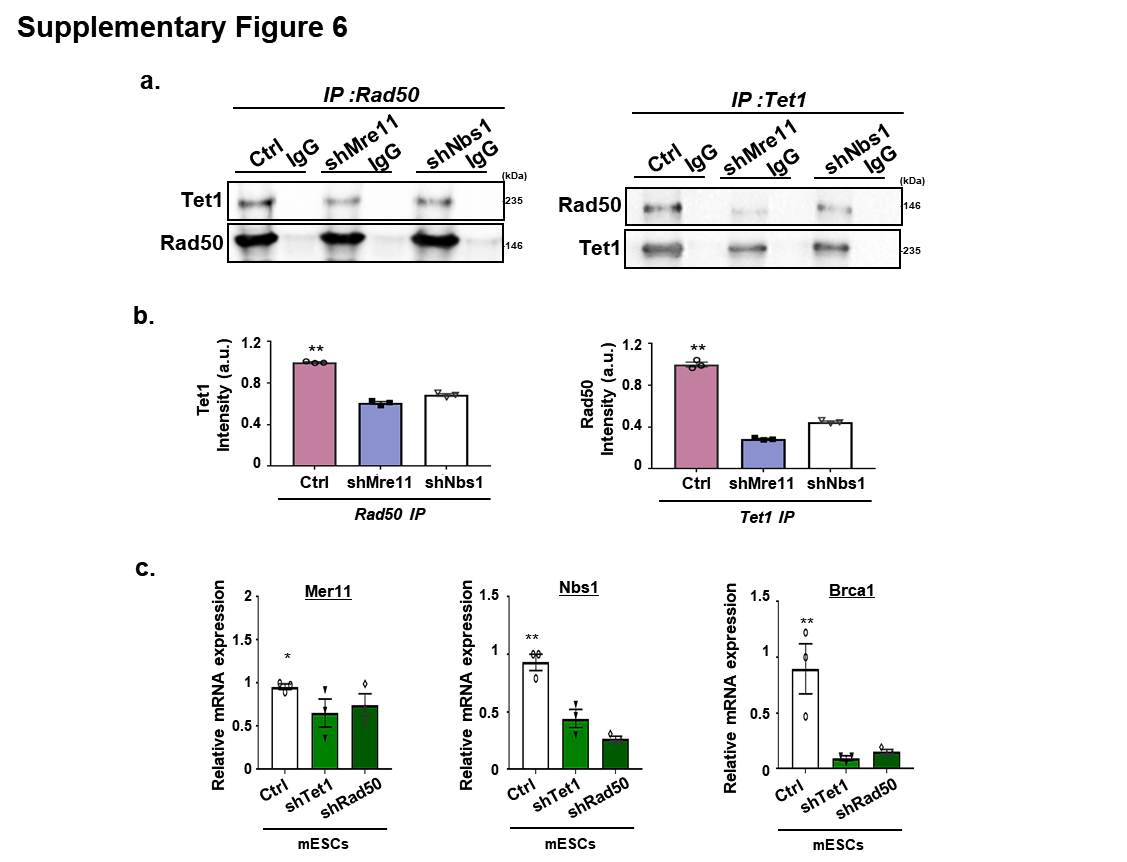
**

**Supplementary Figure 6 DNA repair genes linked to Tet1-Rad50 interaction.** a. Co-IP of Rad50 pulled down with Tet1. Samples treated with DNA repair genes siRNA (Mre11 and Nbs1) were processed for immunoprecipitation with anti-Rad50 and immunoblotted for Tet1. Conversely, samples were processed for immunoprecipitation with anti-Tet1 and immunoblotted for Rad50. d. Quantification of intensities by Co-IP analysis. Data are expressed as the mean ± SEM, n = 3. ***p* < 0.01, ANOVA with Tukey's post-hoc test. c. Quantitative real-time PCR analysis of DNA repair genes (Mre11, Nbs1, and Brca1) after Rad50 and Tet1 knockdown in mESCs. Data are expressed as the mean ± SEM. **p* < 0.05, and ***p* < 0.01, ANOVA with Tukey's post-hoc test.

**Supplementary table 1. List of proteins bound mOct4 promoter sequences identified by LC-MS/MS.**

| **No.** | **Accession** | **Entry** | **Description** | **Mw (Da)** |  |
| --- | --- | --- | --- | --- | --- |
| 1 | P70388 | RAD50_MOUSE | DNA repair protein RAD50 OS Mus musculus GN Rad50 PE 1 SV 1 | 153391 |  |
| 2 | E9QAD7 | E9QAD7_MOUSE | Protein Gm17604 OS Mus musculus GN Gm17604 PE 4 SV 1 | 3453 |  |
| 3 | P02535-2 | K1C10_MOUSE | Isoform 2 of Keratin type I cytoskeletal 10 OS Mus musculus GN Krt10 | 57025 |  |
| 4 | P19426 | NELFE_MOUSE | Negative elongation factor E OS Mus musculus GN Nelfe PE 1 SV 2 | 42528 |  |
| 5 | E9Q1Z0 | E9Q1Z0_MOUSE | Protein 4732456N10Rik OS Mus musculus GN 4732456N10Rik PE 3 SV 1 | 58188 |  |
| 6 | D3YZ77 | D3YZ77_MOUSE | Androglobin Fragment OS Mus musculus GN Adgb PE 2 SV 1 | 85093 |  |
| 7 | Q5SZY7 | Q5SZY7_MOUSE | Protein Prl3d2 OS Mus musculus GN Prl3d2 PE 3 SV 1 | 25509 |  |
| 8 | F6QJ09 | F6QJ09_MOUSE | Protein Gm10203 OS Mus musculus GN Gm10203 PE 4 SV 1 | 7964 |  |
| 9 | B2RY56 | RBM25_MOUSE | RNA binding protein 25 OS Mus musculus GN Rbm25 PE 1 SV 2 | 99491 |  |
| 10 | E9PYI7 | E9PYI7_MOUSE | Protein Lmo7 OS Mus musculus GN Lmo7 PE 1 SV 1 | 164643 |  |
| 11 | Q8BZX4-2 | SREK1_MOUSE | Isoform 2 of Splicing regulatory glutamine lysine rich protein 1 OS Mus musculus GN Srek1 | 69079 |  |
| 12 | J3QPD3 | J3QPD3_MOUSE | Protein Gm8356 OS Mus musculus GN Gm8356 PE 4 SV 1 | 24264 |  |
| 13 | Q99MZ7 | PECR_MOUSE | Peroxisomal trans 2 enoyl CoA reductase OS Mus musculus GN Pecr PE 1 SV 1 | 32389 |  |
| 14 | E9QMY5 | E9QMY5_MOUSE | Protein Shroom3 OS Mus musculus GN Shroom3 PE 1 SV 1 | 214192 |  |
| 15 | F6YZU5 | F6YZU5_MOUSE | Protein Dlg5 Fragment OS Mus musculus GN Dlg5 PE 2 SV 1 | 174469 |  |
| 16 | Q9CUL5 | IQCA1_MOUSE | IQ and AAA domain containing protein 1 OS Mus musculus GN Iqca1 PE 2 SV 2 | 99811 |  |
| 17 | Q6P5D3-2 | DHX57_MOUSE | Isoform 2 of Putative ATP dependent RNA helicase DHX57 OS Mus musculus GN Dhx57 | 149780 |  |
| 18 | O88196 | TTC3_MOUSE | E3 ubiquitin protein ligase TTC3 OS Mus musculus GN Ttc3 PE 2 SV 2 | 223787 |  |
|  |  |  |  |  | |

**Supplementary table 2. List of key hub genes in Rad50 overexpression conditions.**

| **No.** | **Gene Symbol** | **Gene Title** |
| --- | --- | --- |
| 1 | Epc1 | Enhancer of polycomb homolog 1 |
| 2 | Epc2 | Enhancer of polycomb homolog 2 |
| 3 | Ogt | O-linked N-acetylglucosamine (GlcNAc) transferase |
| 4 | Mcrs1 | Icrospherule protein 1 |
| 5 | H3f3a | H3.3 histone A |
| 6 | Brd8 | Bromodomain containing 8 |
| 7 | Usp34 | Ubiquitin specific peptidase 34 |
| 8 | Atrx | ATRX chromatin remodeler |
| 9 | Ccnb1 | Cyclin B1 |
| 10 | Sfpq | Splicing factor proline and glutamine rich |
| 11 | Hist1h2ah | Histone cluster 1 H2A family member H |
| 12 | Zbtb1 | Zinc finger and BTB domain containing 1 |
| 13 | Dnajc2 | Dnaj heat shock protein family (hsp40) member C2 |
| 14 | Bend3 | BEN domain containing 3 |
| 15 | Meaf6 | MYST/esa1 associated factor 6 |
| 16 | Hmgb1 | High mobility group box 1 |
| 17 | Tdg | Thymine DNA glycosylase |
| 18 | Ctcf | CCCTC-binding factor |
| 19 | Ino80c | INO80 complex subunit C |
| 20 | Kdm7a | Ysine demethylase 7A |
| 21 | Nrde2 | NRDE-2, necessary for RNA interference, domain containing |
| 22 | Chd3 | Chromodomain helicase DNA binding protein 3 |
| 23 | H2afz | H2A histone family, member Z |
| 24 | H2afj | H2A histone family, member J |
| 25 | Taf9b | TATA-box binding protein associated factor 9b |
| 26 | Tada1 | Transcriptional adaptor 1 |
| 27 | Pbrm1 | Polybromo 1 |
| 28 | Rybp | RING1 and YY1 binding protein |
| 29 | Coprs | Coordinator of PRMT5 and differentiation stimulator |
| 30 | Mbtd1 | Mbt domain containing 1 |
| 31 | Polr1c | RNA polymerase I subunit C |
| 32 | Polr1b | RNA polymerase I subunit B |
| 33 | Pih1d1 | PIH1 domain containing 1 |
| 34 | Set | SET nuclear proto-oncogene |
| 35 | Kdm8 | Lysine demethylase 8 |
| 36 | Flcn | Folliculin |
| 37 | Tet1 | Tet methylcytosine dioxygenase 1 |
| 38 | Bptf | Bromodomain PHD finger transcription factor |
| 39 | Ing5 | Inhibitor of growth family member 5 |
| 40 | Ing3 | Inhibitor of growth family member 3 |
| 41 | Hcfc1 | Host cell factor C1 |
| 42 | Sirt1 | Sirtuin 1 |
